# Mitophagy in metabolic syndrome

**DOI:** 10.1111/jch.14650

**Published:** 2023-04-11

**Authors:** Mei‐Qi Miao, Yu‐Bo Han, Li Liu

**Affiliations:** ^1^ Department of Cardiovascular The First Affiliated Hospital of Heilongjiang University of Traditional Chinese Medicine Harbin China

**Keywords:** insulin resistance, metabolic dysfunction, metabolic syndrome, MS, mitophagy

## Abstract

Metabolic syndrome (MS), a chronic and non‐communicable pathological condition, is characterized by a constellation of clinical manifestations including insulin resistance, abdominal adiposity, elevated blood pressure, and perturbations in lipid metabolism. The prevalence of MS has increased dramatically in both developed and developing countries and has now become a truly global problem. Excessive energy intake and concomitant obesity are the main drivers of this syndrome. Mitophagy, in which cells degrade damaged mitochondria through a selective form of autophagy, assumes a crucial position in the regulation of mitochondrial integrity and maintenance. Abnormal mitochondrial quality could result in a spectrum of pathological conditions related to metabolic dysfunction, including metabolic syndrome, cardiovascular ailments, and neoplasms. Recently, there has been a proliferation of research pertaining to the process of mitophagy in the context of MS, and there are various regulatory pathways in MS, including pathways like the ubiquitin‐dependent mechanism and receptor‐mediated mechanisms, among others. Furthermore, studies have uncovered that the process of mitophagy serves a defensive function in the advancement of Metabolic Syndrome, and inhibition of mitophagy exacerbates the advancement of MS. As a result, the regulation of mitophagy holds great promise as a therapeutic approach in the management of Metabolic Syndrome. In this comprehensive analysis, the authors present a synthesis of the diverse regulatory pathways involved in mitophagy in the context of Metabolic Syndrome, as well as its modes of action in metabolic disorders implicated in the development of MS, Including obesity, insulin resistance (IR), and type 2 diabetes mellitus (T2DM), offering novel avenues for the prophylaxis and therapeutic management of MS.

## 1 INTRODUCTION

Metabolic syndrome (MS), is a complex medical condition characterized by a cluster of interrelated risk factors, including insulin resistance, high blood pressure, elevated blood sugar levels, excess body fat around the waist, and abnormal cholesterol levels. These factors increase the likelihood of developing serious health problems such as cardiovascular disease and type 2 diabetes. The condition is also commonly referred to as Syndrome X, or insulin resistance syndrome.^1^ The development of metabolic syndrome is primarily driven by over‐consumption of calorie‐dense foods and sedentary lifestyles. These factors result in imbalances in energy intake and expenditure, leading to excess weight gain and accumulation of abdominal fat. This, in turn, increases the risk of insulin resistance and the development of the cluster of health problems associated with metabolic syndrome.^2^ In the United States, MS is estimated to affect 34.7% of adults,^3^ and with the global spread of Western lifestyles, the incidence of MS is increasing dramatically not only in the United States and Europe, but also in Asian countries such as China, Korea, and India,^4^, ^5^ and MS has now become a truly global problem. It is estimated that by 2040 there will be about 2.568 billion people living with MS worldwide.^1^

**FIGURE 1:**
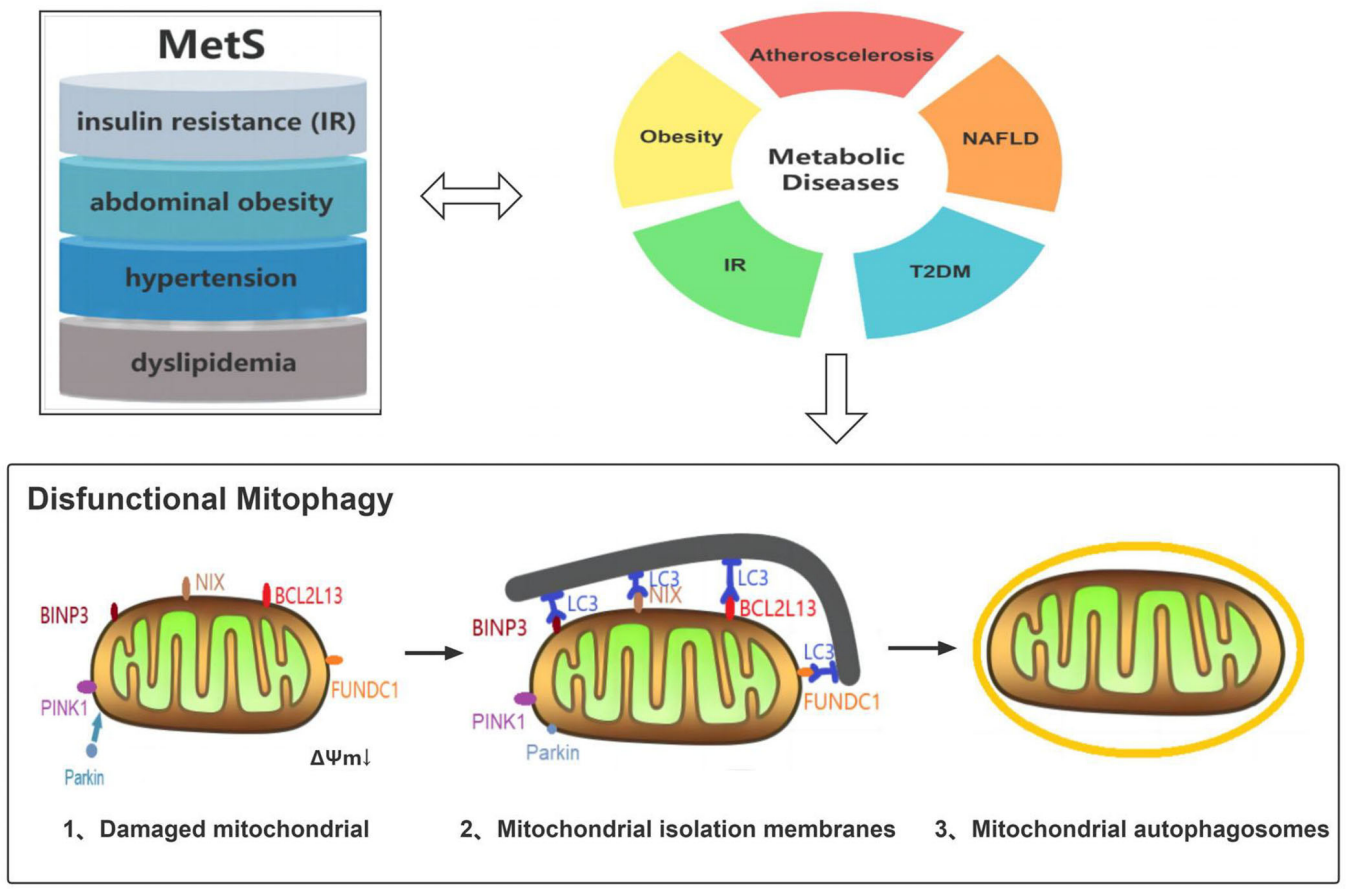
Relationship between mitophagy and MS. MS refers to a conglomeration of metabolic disturbances that encompass overweight, IR, hypertension and abnormal lipid levels. Promotion of mitophagy occurs through specific receptors on the outer mitochondrial membrane or through ubiquitin molecules attached to proteins on the mitochondrial surface, resulting in the formation of autophagosomes surrounding the mitochondria. A growing number of studies have shown that mitophagy has been linked to a range of metabolic disturbances including obesity, IR, T2DM, NAFLD, atherosclerosis (AS), and heart disease, which are pathologically associated with mitophagy dysfunction and may influence the onset of MetS. *Source*: Ref [8].

Autophagy is a cellular degradation pathway that is characterized by its dependence on lysosomes and is widely prevalent in eukaryotic cells as a self‐protective mechanism to maintain a self‐stabilizing intracellular environment.^6^ Mitophagy, a form of macroautophagy, is the process the process of autophagy facilitates the selective removal of damaged mitochondria from cells, which are then transported to lysosomes for subsequent degradation, thereby maintaining mitochondrial homeostasis. Mitophagy is widely recognized as a crucial mechanism of mitochondrial quality control (MQC) and involves three main processes: disruption of the mitochondrial membrane potential, triggering mitochondrial depolarization, and promoting the buildup of mitophagy receptors on the outer mitochondrial membrane (OMM). An increasing body of research has demonstrated a correlation between the cellular process of mitophagy and various metabolic disorders, including obesity,^7^ insulin resistance (IR),^8^ Type 2 diabetes mellitus (T2DM),^9^ non‐alcoholic fatty liver disease (NAFLD),^10^ atherosclerosis (AS), and heart disease,^11^ which are pathologically associated with mitophagy dysfunction and may influence the onset of MS. Therefore, the authors present a comprehensive overview of the underlying mechanisms in the MS and its related metabolic diseases (Figure 1).

## 2 MITOPHAGY PATHWAYS IN MS

Mitophagy can be triggered through a variety of pathways, such as nutritional deficiency (starvation), disruption of the mitochondrial membrane potential, respiratory depression, hypoxia, and ROS accumulation.^12^ Presently, there exist two principal regulatory pathways that regulate the process of mitophagy: The first is mitophagy chiefly overseen by PTEN‐induced putative kinase 1 (PINK1)/cytoplasmic E3 ubiquitin ligase (Parkin) and non‐Parkin‐dependent ubiquitin‐dependent mitophagy; The second refers to mitophagy mediated by mitophagy receptors, that is, non‐ubiquitin‐dependent mitophagy.^13^ These mitophagy receptors are usually mitochondrial proteins containing the microtubule‐associated protein 1 light chain 3 (LC3)/GABARAP‐interacting region (LIR) motif, which can regulates the formation of mitochondrial isolation membranes through specific binding of LIR to members of the mitochondrial Atg8 family (LC3A/B/C, GABARAP, GABARAP‐L1/2). Subsequently, autophagic vesicles selectively encapsulate damaged mitochondria, forming mitochondrial autophagosomes. The degradation of damaged mitochondria within autophagosomes occurs through rapid fusion with lysosomes, thereby promoting the clearance of said mitochondria.^12^ It has been well documented that impaired mitochondrial function is associated with MS and that mitochondrial autophagy leads to impaired mitochondrial function and is involved in the development of MS.^8^, ^14^

### 2.1 Parkin/PINK1

Mitophagy is mainly regulated by the PINK1/Parkin pathway, PINK1/Parkin‐driven mitophagy is the most characteristic pathway and the field of mitophagy is founded on the study of these two proteins.^15^ In normal physiological conditions, PINK1 located in the inner mitochondrial membrane (IMM) is subjected to cleavage and degradation. In the event of mitochondrial damage, a loss of membrane potential impairs IMM translocation and subsequent degradation, leading to the accumulation of PINK1 on the OMM surface where it attracts cytosolic Parkin. Subsequently, the phosphorylation of Ser65 in the ubiquitin and ubiquitin‐like structural domains of Parkin facilitates the localization of PINK1 from the cell membrane to the OMM. Furthermore, Parkin initiates mitophagy through the promotion of mitochondrial fission to isolate damaged fragments, and by ubiquitinating mitochondrial proteins, thereby promoting their recognition and association with the surface of autophagosomes.^16^, ^17^ Hoshino and colleagues demonstrate that PINK1/Parkin pathway‐derived mitophagy protects pancreatic β‐cells and improves glucose intolerance in T2DM patients.^18^ Also, Wang and colleagues found that Long non‐coding RNA H19 exerts a restraining effect on excessive mitophagy by restricting the expression of Pink1 protein, which alleviates this cardiac abnormality that arises during the condition of obesity.^19^ These studies demonstrated that the development of metabolic syndrome (MS) is associated with the PINK1/Parkin pathway‐intervened process of mitophagy.

### 2.2 FUNDC1

FUNDC1 is a newly discovered OMM protein that serves as a receptor for hypoxia‐induced mitophagy. It possesses a characteristic LIR motif and three TM structural domains near the N‐terminal region.^12^ Mutations in the LIR motif of FUNDC1 result in the disruption of FUNDC1‐LC3 interactions and the induction of mitophagy. The function of FUNDC1 is subject to regulation through reversible phosphorylation and ubiquitination, thereby enabling the detachment of damaged mitochondria and the initiation of mitophagy.^20^, ^21^ FUNDC1 regulation is strongly linked to the onset, evolvement, and prognosis of many ailment, including MS. Several experiments have now found that FUNDC1^−^/^−^ mice develop more severe obesity and IR, MS, and myocardial remodelling, and that FUNDC1 deficiency leads to the inhibition of mitochondrial biogenesis, MQC dysregulation and even cell death, suggesting an important role for FUNDC1 in the cardiac function of patients with obese MS.^14^, ^22^

### 2.3 BINP3/NIX

LIR motifs are usually found in mitochondrial proteins that encode mitophagy receptors that recruits LC3 and growing mitochondrial phagocytes into the mitochondria designated to be removed.^23^ Mutations in the N‐terminal region of both BNIP3 and BNIP3L (NIX) block the interaction with LC3 and result in defective mitophagy.^12^ Hypoxia is a relevant trigger for mitophagy, and NIX and BNIP3 mediate post‐hypoxia mitophagy through enhanced activity by phosphorylation and dimerization and transcriptional regulation by hypoxia‐inducible factor‐1α (HIF1α).^15^ It was also found that knockdown of NIX and FUNDC1 resulted in reduced mitophagy during differentiation.^24^ Furthermore, BNIP3 acted upon PINK1 and promotes PINK1 heap up on the OMM and promotes PINK1‐Parkin mitophagy.^15^ A study by Li and colleagues^25^ demonstrated that the downregulation of Sirtuin 3 (Sirt3) inhibited BINP3/NIX pathway‐mediated mitophagy, and activated Sirt3 further activated the ERK‐CREB‐BNIP3 mitophagy pathway, result in the treatment of NAFLD. Activation of BNIP3L caused mitochondrial depolarization, mitophagy, and impaired insulin signaling via the MTOR‐RPS6KB pathway. Furthermore, exercise or pharmacological activation of PRKA, downstream of adrenergic signaling, has been shown to inhibit the function of BNIP3L via mechanisms and restored insulin signaling could overcome IR in myocytes.^26^ These findings suggest the significance of BNIP3 and BNIP3L (NIX) pathways in the regulation of MS‐associated mitophagy, but the exact mechanisms remain to be further investigated.

### 2.4 Others

Bcl‐2‐like protein 13 (BCL2L13), which was recognized as BCL2‐RAMBO, is another OMM protein that is involved in mitochondrial fission in mammalian cells. Li and colleagues^27^ showed the human BCL2L13 protein contains a LIR structural domain, which displays specificity in its binding to three autophagy‐related proteins, namely, LC3C, GABARAP, and GABARAP‐L1 in cells lacking Atg32, which in turn promotes mitophagy. BCL2L13 is significant in controlling apoptosis, mitochondrial fracture, and promoting mitochondrial autophagy.^28^ Ju and colleagues^29^ were the first to publish the novel finding that BCL2L13 promotes the differentiation and facilitation process of adipocytes. Subsequently, a study by Fujiwara and colleagues^30^ found that knockdown of BCL2L13 in 3T3‐L1 adipocytes decreased the number of mitochondria, leading to increased MFN2 protein for mitochondrial fusion, decreased DRP1 protein for mitochondrial division, and enhanced mitophagy, and silencing of BCL2L13 inhibited adipogenic differentiation, mitochondrial biogenesis, and mitochondrial dynamics. Therefore, BCL2L13 plays a role in promoting adipogenesis by enhancing oxidative phosphorylation, preventing apoptosis, and regulating the maintenance of MQC through the process of mitophagy. These fundings suggested that BCL2L13 could be a potential pharmacological therapeutic target for MetS as a promising biomarker.

FK506‐binding protein 8 (FKBP8), which referred to as FKBP38, is another mitochondrial autophagy recognition protein that integrates LC3 independently of Parkin and mediates mitophagy and fission.^31^ Recent study have found that the transmembrane structural domain of FKBP8 needed for its role in starvation‐induced autophagy, and that FKBP8 regulates activation of VPS34 lipid kinase by starvation and autophagy and co‐localizes with complexes of VPS34 lipid kinases and interacts with BECN1.^32^ FKBP8 was found to interact with uncoupling protein 2 (UCP2), which is anchored to the OMM and dynamically regulates insulin secretion from islet β cells and glucose‐stimulated insulin,^33^ and theoretically, FKBP8 is important in the development of IR or T2DM. However, the current research on FKBP8 is limited to cancer and neurological diseases, and the role of FKBP8 in cardiovascular diseases still needs to be further explored.

## 3 MITOPHAGY IN MS

In general, metabolic diseases such as obesity, IR, T2DM, NAFLD, AS, and heart disease lead to abnormal mitochondrial metabolism, which in turn leads to inadequacy β‐oxidation, oxidative stress, and accumulation of toxic lipid antioxidants and mitochondrial dysfunction, and mitochondrial autophagy can treat such metabolic diseases by eliminating this oxidative stress and mitochondrial damage.^34^ In addition, mitochondrial autophagy was found to protect the adipose tissue microenvironment by suppressing obesity‐induced chronic inflammation, excessive oxidative stress, and ER stress,^35^ thereby improving MS. Experiments by Tong and colleagues^7^ showed that HFD‐induced obese mice had mitochondrial dysfunction and heart failure compared to healthy control case. Also, Xiang and colleagues^36^ observed that PINK1/Parkin‐mediated mitochondrial autophagy was activated in submandibular gland cells of diabetic mice. Liu and colleagues^37^ on the other hand showed that quercetin alleviated HFD‐induced improving PINK1/Parkin‐dependent mitophagy as a way to treat hepatic steatosis. In conclusion, mitophagy is related to metabolic diseases like obesity, IR, T2DM, NAFLD, AS, and heart disease, and mitophagy dysfunction is correlated to the etiopathogenesis of these diseases and may influence the pathogenesis of MS.

### 3.1 Mitophagy and obesity

Obesity is a natural consequence of excessive energy intake and sedentary lifestyle, mainly influenced by genetic and environmental factors. Excessive fat deposition in internal organs produces chronic inflammation, which eventually leads to glucolipid abnormalities, hypertension, diabetes, and other complications associated with IR and MS.^38^ It was found that in the muscle of FUNDC1^−^/^−^ mice, LC3‐mediated mitophagy is defective and ATP is reduced, but protective against high‐fat‐diet (HFD) induced obesity, while also improving systemic insulin sensitivity and glucose tolerance.^39^ Lin and colleagues^40^ suggest that sesamol may promote the browning of white adipocytes. Recent studies have found that binding of the nuclear receptors (NR1D1 and ULK1) by which adipocytes are induced to undergo mitophagy, thereby alleviating overweight.^41^ The above studies demonstrate that increased mitophagy may alleviate obesity and that defects in mitophagy are protective against obesity.

### 3.2 Mitophagy and IR

Typically, IR is considered to be the underlying causative factor not only for the metabolic syndrome but also for its associated NAFLD, obesity‐related T2DM and atherosclerotic cardiovascular disease (ASCVD).^42^ Extensive research has shown that mitophagy can improve MQC by increasing ROS production and inhibiting the inflammatory response in adipocytes, thereby inhibiting hepatic IR and steatosis.^8^, ^43^ A recent study found that ginsenoside CK could activate mitophagy in skeletal muscle through the DRP1/PINK1 pathway to maintain MQC, thereby reducing IR in diabetic mice.^44^ In brief, mitophagy can exert its inhibitory effects in IR by maintaining MQC, promoting oxidative stress and inhibiting inflammatory responses, but there are still few studies on the related molecular mechanisms, and a large number of experiments are needed to verify them.

### 3.3 Mitophagy and T2DM

The metabolic stress triggered by T2DM can lead to pancreatic β‐cell dysfunction and IR,^45^ and likewise, these contribute to MS. It has been found that there are many natural products that regulate mitophagy such as curcumin, caffeine, quercetin, berberine, and vitamins that can improve mitochondrial dysfunction related to T2DM.^45^ The correlation of T2DM and ischemia‐reperfusion is inextricably linked, and adiponectin was found to attenuate the damage caused by reduced blood flow followed by its restoration, leading to an increase in oxidative stress, inflammation, cell death, and disrupted function of the mitochondria in lung‐injured tissues of T2DM mice by activating SIRT1‐ PINK1 signaling‐mediated mitohagy.^46^ Furthermore, a recent study found that myocardial ischemia‐reperfusion (MIR) exacerbates the extent of damage in T2DM by inhibiting mitophagy. MitoQ, a mitochondria‐targeted antioxidant, can exert a protective effect on cardiomyocytes after MIR in T2DM by increasing its expression level through PINK1/Parkin pathway‐mediated mitophagy.^47^ These conclusions demonstrate that mitochondrial autophagy participates in the development of T2DM through attenuated mitochondrial dysfunction, inflammation, and oxidative stress.

### 3.4 Mitophagy and NAFLD

NAFLD is the development of steatosis of the liver, whether had inflammation, fibrosis or not, lack of confounding factors such as excessive alcohol intake that may contribute to secondary liver lipid accumulation. Primary harmful index, for example, obesity, T2DM, and dyslipidemia; therefore, it can be considered a hepatic manifestation of the MS.^48^ In recent years, a new concept called “metabolic (dysfunction) associated fatty liver, MAFLD” has even emerged to describe the relationship between NAFLD and metabolic dysfunction, emphasizing the simultaneous presence of lipid deposition in liver and abnormal metabolic function.^49^ Zhou and colleagues^50^ showed that the NR4A1/DNA‐PKcs/p53 pathway emerged as a novel molecular circadian mechanism leading to NAFLD, NAFLD by regulating mitophagy related to Bnip3 and Drp1, and also confirmed that melatonin stopped mitochondrial fission by interdicting the NR4A1/DNA‐PKcs/p53 pathway and restored mitophagy, finally improving mitochondrial and hepatic feature in NAFLD patients. Furthermore, PINK1‐mediated mitophagy might be responsible for CYANID‐3‐O‐glucoside's facilitation on NAFLD.^51^ The latest study found that deletion of selenoprotein M (SELENOM) exacerbated HFD‐induced the presence of steatosis, inflammation, and fibrosis in the liver in obese mice, an increase in fatty acid oxidation and oxidative stress in the liver is associated with this condition; over‐expression of SELENOM activated Parkin‐mediated mitophagy through the AMPKα1‐MFN2 pathway, which cleared HFD‐damaged mitochondria.^52^ Mitophagy may combat MS by improving mitochondrial dysfunction, and liver function in patients with NAFLD.

### 3.5 Miotophagy and AS

In atherosclerosis, lipids and leukocytes heap up in artery due to chronic inflammation, leading to plaque formation. MS was related to metabolic, pro‐inflammatory, and pro‐thrombotic states characterized by a series of interrelated plaque buildup influencing index, including OS, dyslipidemia, hypertension, hyperglycemia, obesity, IR, and living habit, containing diet structure and lack of exercise, that can increase the risk of atherosclerotic injury.^53^ A study by Ma and colleagues^54^ found that melatonin could scavenge ROS and attenuate the activation of NLRP3 (nucleotide‐binding domain and leucine‐rich repeat pyrin domain containing 3) inflammatory vesicles through the Sirt3/FOXO3a/Parkin mitophagy pathway in macrophages, which in turn significantly attenuated the size and vulnerability of AS plaques for the purpose of treating AS. In addition, Chen and colleagues^55^ also summarized the various regulatory mechanisms of mitophagy in AS, including various cytokines, thrombi, and senescence. Thus, it is evident that mitophagy participates in AS pathogenesis and may be a novel therapeutic target.

## 4 SUMMARY AND OUTLOOK

MS is characterized by the coexistence of obesity, hypertension, dyslipidemia and hyperglycemia in individuals, causing the higher risk of CVDs. This paper focuses on the various cellular pathways of mitophagy in MS, such as the classical PINK1/Parkin pathway, the mitophagy receptor FUNDC1, and the BINP3/NIX pathway, as well as the regulatory mechanisms of mitophagy in MS‐related metabolism‐related illness like obesity, IR, DM, NAFLD, AS, and heart disease. Therefore, mitophagy may serve as a new target for the prevention and arrangement of MS, and an in‐depth investigation of the relationship between mitophagy and MS required fully understand the significance of mitophagy in MS. The current study found that mitophagy basically regulates the development of MS through anti‐inflammation and anti‐oxidative stress, and so on. On this basis, a more in‐depth investigation of the specific mechanisms of FKBP8 and BCL2L13‐related mitophagy pathways in MS‐related metabolic diseases such as IR and T2DM can help to understand the role of mitophagy in MS more comprehensively and thorough.

## AUTHOR CONTRIBUTIONS

Mei‐Qi Miao contributed to the conception of the article, and wrote the manuscript. Yu‐Bo Han contributed significantly to manuscript preparation, and the review and revision of the original draft. Liu Li provided funding for this review.

## CONFLICT OF INTEREST STATEMENT

The authors affirm the absence of any competing interests.

## Data Availability

None.
